# Aptamer-conjugated live human immune cell based biosensors for the accurate detection of C-reactive protein

**DOI:** 10.1038/srep34778

**Published:** 2016-10-06

**Authors:** Jangsun Hwang, Youngmin Seo, Yeonho Jo, Jaewoo Son, Jonghoon Choi

**Affiliations:** 1 School of Integrative Engineering, Chung-Ang University, Seoul 06974, Republic of Korea

## Abstract

C-reactive protein (CRP) is a pentameric protein that is present in the bloodstream during inflammatory events, e.g., liver failure, leukemia, and/or bacterial infection. The level of CRP indicates the progress and prognosis of certain diseases; it is therefore necessary to measure CRP levels in the blood accurately. The normal concentration of CRP is reported to be 1–3 mg/L. Inflammatory events increase the level of CRP by up to 500 times; accordingly, CRP is a biomarker of acute inflammatory disease. In this study, we demonstrated the preparation of DNA aptamer-conjugated peripheral blood mononuclear cells (Apt-PBMCs) that specifically capture human CRP. Live PBMCs functionalized with aptamers could detect different levels of human CRP by producing immune complexes with reporter antibody. The binding behavior of Apt-PBMCs toward highly concentrated CRP sites was also investigated. The immune responses of Apt-PBMCs were evaluated by measuring TNF-alpha secretion after stimulating the PBMCs with lipopolysaccharides. In summary, engineered Apt-PBMCs have potential applications as live cell based biosensors and for *in vitro* tracing of CRP secretion sites.

The characterization and identification of interactions between cells and biomolecules provides a significant basis for understanding the origin, progress, and prognosis of human diseases. Biological interactions include protein-to-protein and antigen-to-antibody reactions. Recent immunochemical techniques have helped explain unknown phenomena mediating antigen and antibody reactions, and their high sensitivity and selectivity have made them popular diagnostic tools. Immunochemical techniques, however, depend on the quality of antibodies for their sensitivity and specificity, which limits their applications^1^,^2^.

Recent advances in the preparation of aptamers have promoted their usage in place of antibodies due to their comparable binding affinities and stability under heat or pH variation^3^,^4^. In addition, aptamers can be easily synthesized, isolated, and modified, and are highly resistant to denaturation. Single-stranded DNA (ssDNA) or RNA aptamers bind to a specific domain of a target protein, and aptamers conjugated with fluorescent dye molecules are employed for biosensing specific targets using various array platforms^5^,^6^,^7^,^8^. Aptamers have been adopted as *in vitro* sensing probes; however, their application has been limited to *in vivo* experiments due to the difficulty of choosing a proper delivery vehicle (liposome, nanoparticle, cell, etc.) for them^9^,^10^.

C-reactive protein (CRP) is a ring-shaped, pentameric protein produced in the liver; it increases in the serum upon infection or immunological response, and is especially upregulated in the case of cardiovascular disease^11^,^12^. CRP binds to phosphocholine expressed on the surface of dead cells and activates a complement system promoting phagocytosis. Acute-phase immune responses increase the level of interleukin 6 (IL-6) in the peripheral blood and eventually upregulate CRP production in the liver^13^,^14^. Therefore, the quantification of CRP in the bloodstream could provide an important marker for diagnosing bacterial or virus infections and associated tissue degeneration^15^,^16^. The normal level of CRP in the bloodstream is less than 3 mg/L, but it can reach up to 500 times that number in the blood of patients with cardiovascular disease^17^. One common immunochemical technique for CRP detection is enzyme-linked immunosorbent assay (ELISA), which has a limit of detection of 0.5–1.0 μg/L. A recent study in which laser nephelometry was utilized for a CRP test detected levels as low as 0.04 mg/L^18^. Since the study focuses on the evaluation of live cell based sensors for their sensitivity in detecting the fewest amount of CRP, we prepared the CRP standards in their concentration ranges from 0.01 to 30 mg/L, which covers the usual ranges of CRP concentrations specific for heart disease or inflammations (1–10 mg/L)^11^.

In this study, we prepared CRP-specific, aptamer-conjugated human peripheral blood mononuclear cells (Apt-PBMCs) to evaluate their use as live cell based biosensors (Fig. 1). A series of conjugations, including biotin-streptavidin affinity, was employed for the successful and biocompatible linkages between live blood cells and aptamers.

Peripheral blood mononuclear cells (PBMCs) are non-attaching, non-differentiating, independent immune cells (e.g. T, B cells) without stimulation that participate in innate or acquired immune responses in the human body. Among PBMCs, lymphocytes are key players for cell-cell immunity and are able to migrate to sites of inflammation^19^,^20^. By introducing aptamers to PBMCs, we designed a method to visualize their homing mechanisms and *in vivo* trafficking. Engineered live cell sensors have advantages for the direct observation of functional crosstalk between cells and biomolecules as well as monitoring continuous changes in surrounding microenvironments. In addition, live cell sensors may serve as reporters for the high-throughput screening of multiple drug molecules^21^.

We evaluated the properties of Apt-PBMCs as live cell biosensors and their function as a group of immune cells that properly respond to external stimuli. Apt-PBMC complexes can serve as modular platforms in which the target-specific aptamer can be replaced, allowing switchable and specific biosensing of multiple antigens. They may also be beneficial for the spontaneous sensing and monitoring of micro-environmental changes as infectious diseases progress. Since the goal of this study is to engineer engineering aptamer and live cell complexes as well as to evaluate the PBMCs’ potential as a novel live cell based sensor for specific target molecules, we focused on the fundamental development of bioconjugation approaches and optimization of the processes. We anticipate the preparation of Apt-PBMCs would serve as a stepping stone to construct biocompatible, autologous, non-immunogenic live cell based sensor systems for simultaneous detection of targets including disease biomarkers, inflammation sites, or cancerous cells, etc. In order to use the live cell based sensor systems *in vivo*, however, the further development of current system is required. The detection of target molecules should be possible without the needs of secondary antibodies, for example employing aptamer beacons that can switch on or off their signals upon coupling with target molecules.

## Methods

### Materials and Methods

Streptavidin, the Mix-n-Stain antibody labeling kit, and calcium chloride were purchased from Sigma (St. Louis, MO, USA). The sulfo-NHS-biotin labeling kit and well plates were purchased from Thermo Scientific (Waltham, MA, USA). CRP standard protein, anti-human CRP monoclonal antibody, and the human TNF-alpha ELISA kit were purchased from R&D Systems (Minneapolis, MN, USA). The human C-reactive protein (CRP) ELISA kit was purchased from eBioscience (San Diego, CA, USA). Phosphate-buffered saline (PBS), DPBS without Ca^2+^ and Mg^2+^, and PRMI 1610 medium were obtained from Gibco (Waltham, MA, USA). All reagents were dissolved in ion-free water or PBS without Ca^2+^ and Mg^2+^ (PBS-), and calcium ions were continually detected/checked throughout the experiment.

### Design of ssDNA aptamers

According to the previous report elsewhere, the range of aptamer and CRP binding affinity is from 0.3 to 30 nM^22^. The ssDNA aptamers consisted of 72-mers with the following sequences:

5′-Biotin-GGCAGGAAGACAAACACACAAGCGGGTGGGTGTGTACTATTGCAGTATCTATTCTGTGGTCTGTGGTGCTGT-FAM-3′, and 5′-Biotin-GGCAGGAAGACAAACACACAAGCGGGTGGGTGTGTACTATTGCAGTATCTATTCTGTGGTCTGTGGTGCTGT-3′. The 5′ ends of aptamers were conjugated with biotin and the 3′ end of one of the aptamers was modified with 5-FAM (5-carboxyfluorescein) fluorescent dye to confirm successful conjugation on cells.

### Preparation of PBMCs

Human PBMCs were obtained from CTL (Cellular Technology Limited, Shaker Heights, OH, USA). Cell culture media consisted of RPMI 1640 (Gibco, Life Technologies) supplemented with 1% penicillin/streptomycin and 10% fetal bovine serum. Media were passed through 0.2-μm (pore size) syringe filters. The PBMCs were rested for 16 h in 5% CO_2_ at 37 °C after thawing.

### Preparation of Apt-PBMCs

Rested PBMCs were washed with DPBS by spinning down the cells (200 × *g*, 7 min) and re-dispersing the pellet. Washed PBMCs were tested for viability and counted to obtain more than 1 × 10^7^ live cells/mL. Washed PBMCs were re-dispersed in 400 μL of DPBS supplemented with 1 mM sulfo-NHS-biotin and maintained for 30 min at room temperature. After the reaction, cells were spun down (200 × *g*, 7 min) and the supernatant was discarded. After two more washes with DPBS, the cell pellet was suspended in 400 μL of DPBS supplemented with 50 μg/mL streptavidin. After a 15-min reaction at room temperature, the cells were washed twice with filtered, serum-free RPMI 1640. Next, 1 μL of 100 pM biotin-conjugated aptamer was dissolved in DPBS (with Ca^2+^ and Mg^2+^) at 1:10 v/v. The solution was then heated at 95 °C for 5 min to obtain ssDNA aptamers and abruptly cooled to 4 °C for 10 min to maintain the ssDNA structure. The cell pellet was suspended in 390 μL of serum-free RPMI 1640 with 10 μL of single-stranded biotin-conjugated aptamer solution. After a 15-min reaction at room temperature, the cells were washed three times.

### Confocal microscope images

Confocal microscopic images of the cells were obtained using the Carl Zeiss LSM 710 laser scanning microscope (Carl Zeiss, Oberkochen, Germany). Apt-PBMC complexes that captured CRP were stained using an anti-hCRP antibody with dye molecules (Mix-n-Stain kit, λ_*ex*_/λ_*em*_ = 593/614 nm). The CRP-captured Apt-PBMC pellet was delivered to 50%, 70%, and 100% ethyl alcohol solutions in a stepwise manner for dehydration and fixation. Completely fixed cells were air-dried and washed with PBST (PBS with 0.05% Tween 20) twice. Next, 1 mL of DAPI working solution (4′,6-diamidino-2-phenylindole) was added to stain the nuclei of the fixed cells. The cells were completely dried after another wash with PBST.

### Confirmation of continuous coupling

To validate the bioconjugation steps, magnetic beads were chosen to serve as cell-carrying aptamers. Apt-magnetic bead complexes were prepared using EDC/NHS cross-linkers, connecting biotinylated anti-hCRP antibodies to the carboxylated magnetic beads (M270: the mean diameter of beads is 2.8 μm comparable to that of non-stimulated immune cells). Heat treated aptamer was introduced to streptavidin/beads and 1% BSA was added to prevent any unwanted binding on the surface. Recombinant CRP was diluted at concentrations of 4.2, 8.4, 16.8, and 25.2 μg/L in DPBS and reacted with apt-magnetic bead complexes (The concentrations of standards were chosen to probe the minimum of detectable fluorescence from a magnetic bead capture). The fluorescence signal was detected by adding fluorescent dye labeled anti-hCRP antibody (λ_*ex*_/λ_*em*_ = 590/620 nm) to the complexes.

### TNF-alpha profiling assay

PBMCs and Apt-PBMCs were seeded in 24-well plates at a density of 1.5 × 10^5^/well and stimulated with 0.2 μg/mL lipopolysaccharides (LPS). After 24-h incubation, the secretion of TNF-alpha by cells was quantified using ELISA (human TNF-alpha ELISA kit, R&D Systems).

### Cell viability assay

Cell viability was measured using a trypan blue assay. Apt-PBMCs and naïve PBMCs were cultured for 3 d at 37 °C and 5% CO_2_. Every 12 h, the cells were spun down and washed for trypan blue staining. The numbers of live cells and dead cells were counted using a cell-counting instrument (JuLI Br; Invitrogen, Carlsbad, CA, USA).

### Preparation of the CRP standard protein

Recombinant CRP was dissolved in DPBS (pH 8.0 with 0.2% BSA). CaCl_2_ was added to 50 μL of the CRP solution to a concentration of 10 mM, and the solution was maintained for 1 h to allow for the complete reaction. Unbound calcium ions were isolated using an ultrafiltration kit (Mix-n-Stain kit, Sigma) by centrifugation at 14,000 rpm for 5 min.

### Apt-PBMC **capturing** assay

On an epoxy-terminated glass, simple channels were drawn using an organic marker, and the standard solutions of recombinant CRP (0, 4, and 4 mg/L) dissolved in a printing buffer were separately dropped onto the channel at 4 °C. The channel was blocked with 3% BSA for 1 h at 37 °C and then washed twice with BPST and water. Next, DAPI-stained Apt-PBMCs were dispersed in PBS (20 μL) and 1 × 10^7^ cells/mL were loaded on a channel for the capturing reaction. After the channel was washed with PBS and water, the attachment potentials of Apt-PBMCs were monitored.

In order to mimic the blood vessel, we employed a designed microchannel (400 μm × 50 μm), which have flow speed of 0.01 μm/min and made of PDMS mixture. Plasma-treated PDMS chip on a glass slide was later spotted with a 0.1μL of CRP standard solution (30 mg/L) by using a microarray spotter (Arrayit SpotBot^®^ Extreme Microarray Spotters). Prepared PDMS microchannel was flown by a heparinized, diluted (1:10,000) sheep blood containing 5 × 10^6^/mL of Apt-PBMCs (Supporting Fig. 2). To visualize Apt-PBMCs attaching to the CRP spots, aptamers modified with FAM were treated on the cells as well as the DAPI staining to the nucleus of cells.

## Results and Discussion

CRP has a homopentamer structure consisting of 224 amino acids and requires Ca^2+^ as a cofactor that binds to each subunit. The cofactor has effects on the formation of CRP and sensitivity of antibodies and aptamers^23^. (Supporting Fig. 1B). Although the addition of calcium ions to the cell culture may increase the sensitivity of CRP detection, it also promotes the lysis of cells. Accordingly, controlling the optimal amount of calcium ions in the cell culture and reagents is a key factor in CRP detection using Apt-PBMCs.

### Confirming each molecule for construction of the cell based sensors

To prove our hypothesis that each PBMC may function as a cell sensor, we introduced magnetic beads as cell cores. Introducing EDS/NHS to the carboxylic group on the surfaces of magnetic beads provided a moiety to biotinylated anti-CRP antibodies. After conjugating magnetic beads and streptavidin, biotinylated aptamer was introduced. Various concentrations of CRP were evaluated to determine concentration-dependent capture by the formation of magnetic bead/aptamer/CRP complexes. To confirm the binding formation of the designed aptamer-complex, dye-labeled CRP antibody was employed. It should be noted that the presence of biotinylated aptamer would be an essential key to confirm the effectiveness of selected aptamers for detecting CRP (Fig. 2). As low as 8.4 ng/mL concentration of recombinant CRP was detected in our system. As the amount of CRP increased, the fluorescent intensity also increased. Fluorescence from the control that did not contain aptamers indicates the binding of streptavidin PE to the biotin regions on magnetic bead/biotinylated antibody complexes which indirectly proved biotin-streptavidin interaction as well as signal indicator for magnetic bead/antibody/CRP complexes. In the control samples (i.e. the absence of aptamers or CRPs in the complexes), there were a significant decrease of fluorescence intensities, which validate the success of our bioconjugation approach.

Our system was based on sandwich immunoassay, which requires fluorescence-labeled antibodies or beads as reporters. The fluorescence-labeled antibodies would also help to confirm the successful capture of CRP using Apt-PBMCs in the final complexes. The concentration-dependent change in fluorescent intensity enabled the potential of our Apt-PBMC system for sensor applications and demonstrated a success of bioconjugation steps. In addition, Apt-magnetic beads were verified as a cell core, which has applications for the *in vitro* probing of biomolecules.

### Preparation of the Apt-PBMCs complex

**Figure 1:**
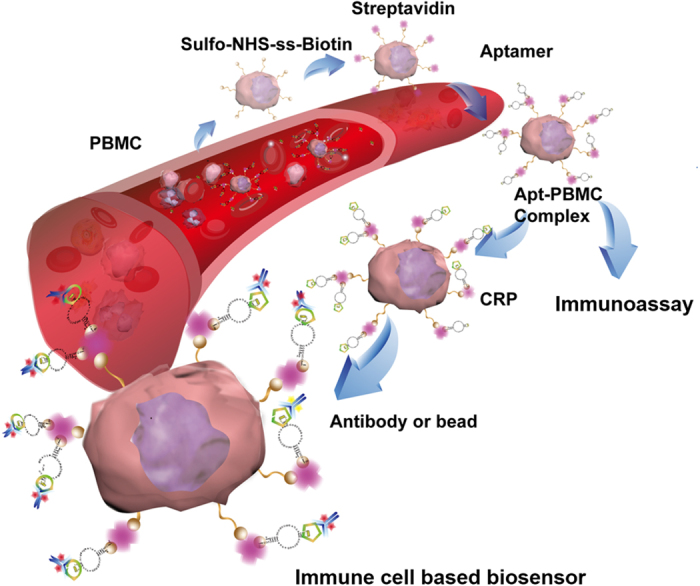
Illustration of aptamer-conjugated PBMCs for the detection of CRP molecules. Sulfo-NHS-SS-biotin was conjugated to PBMCs by a crosslinking reaction, followed by the introduction of streptavidin to combine with biotin. Next, the biotinylated-aptamer was linked to the complex, forming aptamer-conjugated PBMCs (Apt-PBMCs). The complex migrates in the fluid and recognizes CRP, forming a CRP-Aptamer-PBMC complex. Finally, the anti-CRP antibody or antibody coated-beads were attached to the conjugated complex, emitting a detectable florescence signal.

**Figure 2:**
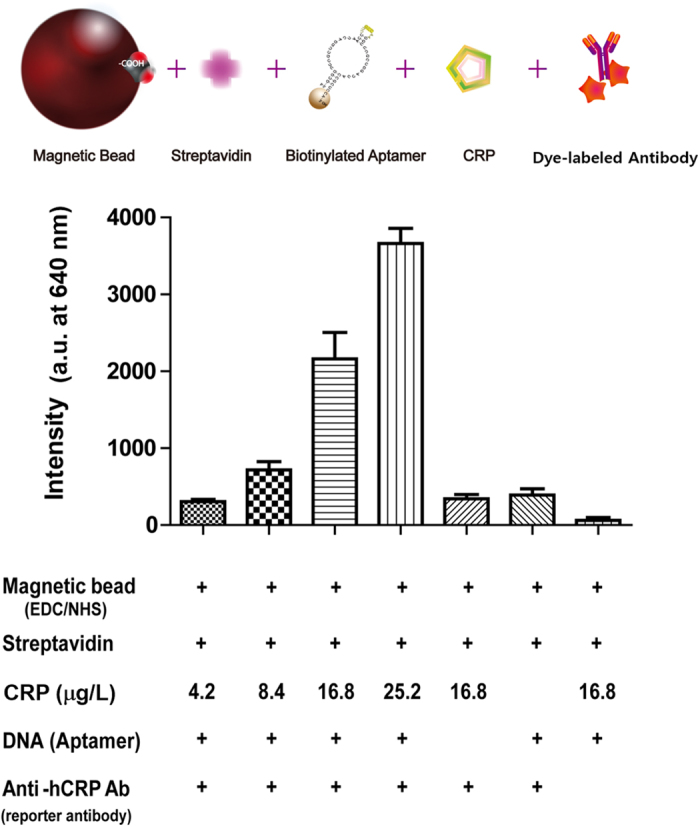
Confirmation of the bioconjugations and the function of aptamer, CRP and antibody. CRP detection was measured in the presence or absence of aptamers for different CRP concentrations and the evaluation of each step was performed in the presence or absence of the anti-hCRP Ab. Carboxylated magnetic beads were used instead of cells (Size of beads are 2.8 μm), and streptavidin PE was used as a signal indicator. (CRP concentration = 4.2, 8.4, 16.8 and 25.2 μg/L, control indicates the absence of antibody, aptamer and CRP protein in the complexes, and 1% bovine serum albumin were used as blocking buffer, λ_ex_/λ_em_ = 590/620 nm (n = 5).

**Figure 3:**
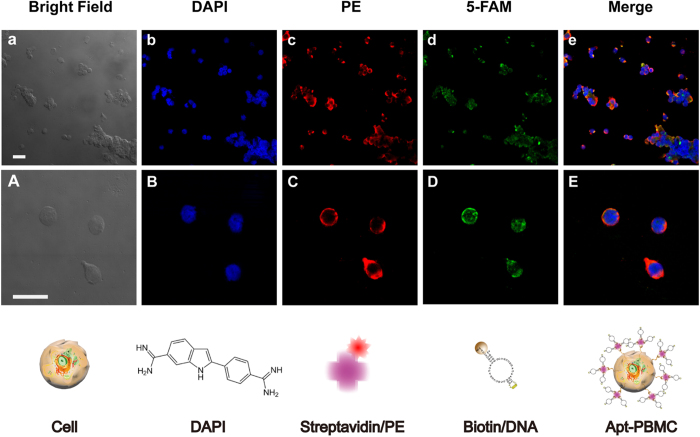
Fluorescence images of Apt-live cell complexes. (**A**) Bright-field images of PBMCs; (**B**) DAPI (4′,6-diamidino-2-phenylindole)-stained PBMCs; (**C**) Sulfo-NHS-SS-biotin-linked PBMCs conjugated with streptavidin PE (phycoerythrin); (**D**) 5-FAM-labeled and biotinylated aptamers on PBMCs; (**E**) Apt-PBMC live cell complexes (merged images). Cell count = 1.5 × 10^5^/slide, scale bar = 20 μm. (**a–e**) low-resolution images of cells, (**A–E**) high-resolution images of individual cells for each step of bioconjugation).

Figure 1 summarizes the process by which Apt-PBMC complexes were prepared. Each conjugation step was confirmed by measuring fluorescence signals using dyes tagged to the ligands of each conjugate. The dispersant of PBMCs (92% viability, 1 × 10^7^/mL) was mixed with sulfo-NHS-biotin, which covalently binds to the amine groups of surface proteins on PBMCs via its NHS ester, forming an amide linkage and releasing *N*-hydroxysuccinimide. To minimize the potential toxicity of the reaction process, the total reaction time was limited to 30 min and the cell pellet was washed three times with serum-free RPMI1640. Serum-free RPMI1640 confers stability to the PBMCs during bioconjugation processes. To confirm the successful modification of the cell surfaces, 1/200 diluted streptavidin PE (5 μL/tube) was added to the cell-crosslinker conjugates and PE fluorescence was measured (Fig. 3C, λ_*ex*_/λ_*em*_ = 500/580 nm). PE on cell conjugates resulted in red fluorescent cells, as shown in Fig. 3. Next, ssDNA aptamers modified with biotin at the 5′ end were introduced to the cell conjugates. Calcium chloride and magnesium chloride containing PBS were used as solvents to provide positive ions essential for DNA structural confirmation. Given that ion-containing PBS may cause problems with cell handling, we optimized the conditions for the conjugation process (Supporting Fig. 1A). To confirm the successful conjugation of aptamers to the cell conjugates, we modified the 3′ ends of aptamers with 5-carboxyfluorescein (5-FAM) and measured the fluorescence of the product (Fig. 3D). In addition, we stained the cell nuclei with DAPI to verify PBMCs with ligands attached to the cell surfaces.

### Detection of CRP

**Figure 4:**
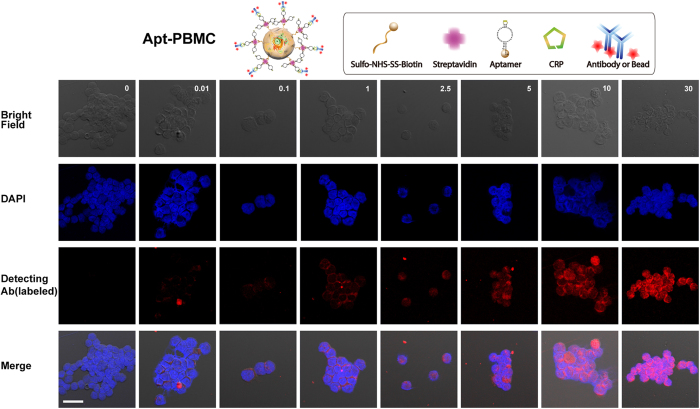
Florescence images of PBMCs capturing CRP. Bright-field images of Apt-PBMCs, DAPI-stained Apt-PBMCs, Apt-PBMCs are capturing CRP which visualized labeled fluorescence antibodies, and merged images (from top to bottom). Apt-PBMCs/CRP were stained with a florescent dye (λ_ex_/λ_em_ = 593/614 nm); PBMCs: 1 × 10^5^/slide; Scale bar = 20 μm.

**Figure 5:**
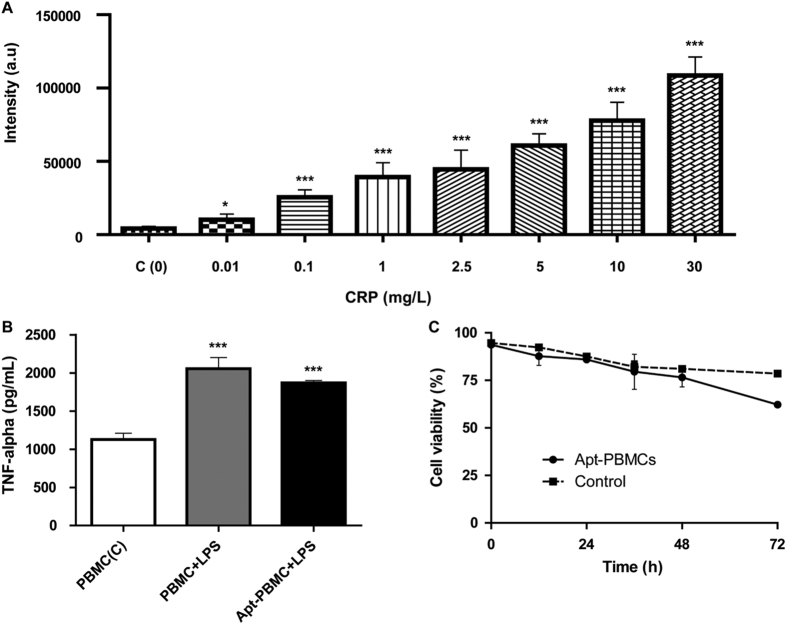
Characteristics of Apt-PBMCs and their concentration-dependent ability to sense CRP. (**A**) Levels of fluorescence intensity from Apt-PBMCs capturing CRP at different concentrations. Cell count was 1 × 10^5^ per test; 15 cells were evaluated by image analysis (***P-value < 0.0001, *P-value < 0.05). (**B)** TNF-alpha profiling assay. Both PBMCs and Apt-PBMCs were seeded (1 × 105 per well) and incubated for 24 h after adding LPS (0.2 μg/mL) (***P-value < 0.0001). (**C**) Cell viability curve for Apt-PBMCs. Both PBMCs and Apt-PBMCs were cultured for 72 h and a trypan blue assay was used to count viable cells (seeding density = 1.5 × 105/well).

The Apt-PBMC complexes were confirmed by confocal microscopy (Fig. 3E), and their CRP-capturing ability was examined. A dilution series of recombinant CRP was prepared and reacted with Apt-PBMC complexes for capture prior to sandwich immunoassay with anti-CRP antibodies labeled with a fluorescent dye. Apt-PBMC complexes were prepared in tubes at a cell concentration of 4 × 10^5^/mL and recombinant CRP was added to each tube at concentrations of 0, 0.01, 0.1, 1, 2.5, 5, 10, and 30 mg/L. Since the lower limits of detecting CRP using the magnetic bead system were not applicable to the Apt-PBMCs, we scaled up the amounts of target CRP spanning the concentrations from 0 to 30 mg/L that are comparable to those of CRP in a physiological condition. Increasing concentrations of CRP elevated the fluorescence intensity due to increased capture by the Apt-PBMC complexes(Fig. 4). The CRP capture affinity of Apt-PBMC complexes was dependent on the concentration of positive ions and the type of buffer (Supporting Fig. 1)^23^. We optimized the protocol to minimize cell damage while maximizing fluorescence intensity. It has been reported that the serum contains about 9 mM of Calcium ions and about 10 mM of Calcium ions per day transports between bones and extracellular fluid (ECF). Since Calcium ions are used as a cofactor for CRP, we added comparable amount of Calcium ions to recombinant CRP solution to mimic a physiological environment^23^,^24^. CaCl_2_ (10 mM) was added to the CRP solution before the reaction and the CRP/Ca^2+^ conjugates were isolated by filtering out the extra calcium ions, BSA, and other components of the solution. Equal amounts of Apt-PBMC complexes were evaluated to compare the amounts of CRP captured with and without added Ca^2+^. The fluorescence intensity was at least 20 times stronger for CRP/Ca^2+^ than for CRP without Ca^2+^ (Supporting Fig. 1B). Proteins in the serum conjugated with Apt-PBMC complexes would result in false positive results and require thorough filtration. The average intensity of 15 individual Apt-PBMCs was calculated to measure the concentration of captured CRP (Fig. 5A). The limit of detection was 0.05 mg/L.

### TNF-alpha profiling

TNF-alpha is a cytokine involved in inflammatory responses; it is secreted from macrophages or T cells and modulates the production of IL-1 and IL-6 as well as controls cell-mediated immune responses via apoptosis^25^. To confirm the integrity of Apt-PBMCs as an immune response mediator, we added LPS (0.2 μg/mL per well) to the Apt-PBMC culture and measured the production of TNF-alpha. LPS-stimulated Apt-PBMCs produced ~90% of the TNF-alpha secreted by naïve PBMCs, which validated the functional integrity of the aptamer-modified PBMCs (Fig. 5B). The amounts of sulfo-NHS-biotin and streptavidin used in our work were optimized to minimize damage to the normal function of PBMCs.

### Cell viability test

Cell viability before and after the series of conjugations is a critical parameter for their use as live cell based sensors. We evaluated the viability of control PBMCs and Apt-PBMC complexes for 72 h after their synthesis (Fig. 5C). The viability of Apt-PBMCs remained the same for 36 h after preparation and decreased gradually thereafter. The viability of Apt-PBMCs was maintained at up to 65% after 3 day post-preparation; accordingly, they are promising live cell based sensors for *in vitro* applications that require high sensor stability.

### Apt-PBMC binding specificity assay

**Figure 6:**
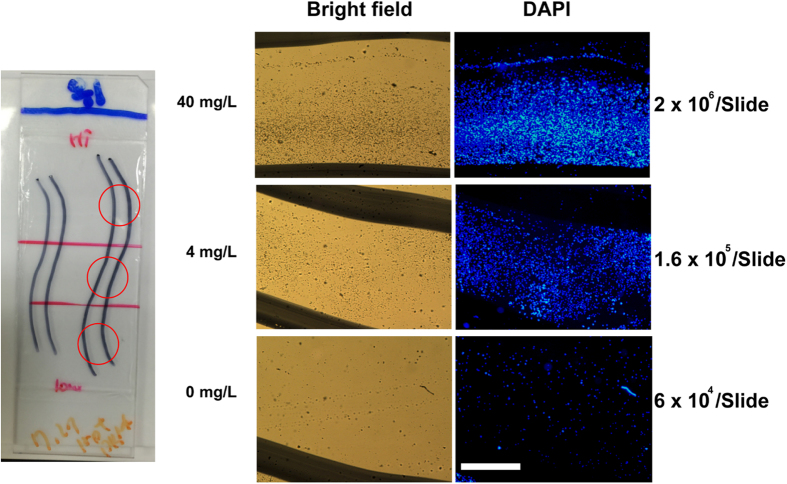
Apt-PBMC binding specificity assay. A hydrophobic barrier was drawn on an epoxy glass slide to generate a 3 mm-wide channel. Three sections of the glass slide were coated with three different concentrations of CRP. Apt-PBMCs were flown through the channel and the channel was washed to measure the binding patterns of Apt-PBMCs. (1 × 10^7^ /mL Apt-PBMCs; DAPI staining of cells was used for visualization; scale bar = 1 mm).

In order to evaluate the binding of Apt-PBMCs to the sensing target molecules, we prepared a simple, 3 mm-wide channel on an epoxy glass slide by drawing arbitrary curves with a permanent marker pen (Fig. 6). The inner side of the channel surface was immobilized with various concentrations of recombinant CRP (0, 4, and 40 mg/L on each section). After Apt-PBMCs flowed through the channel, captured Apt-PBMCs were observed on the surface of the glass slide via conjugation with surface-immobilized CRP. DAPI staining of the cells showed a binding pattern of Apt-PBMCs in which the section with a higher concentration of CRP contained more cells (2 × 10^6^) (Fig. 6). Other sections of the channel exhibited 1.6 × 10^5^ and 6 × 10^4^ captured cells/slide, confirming that Apt-PBMC specific binding was concentration-dependent.

Figure 6 demonstrates the positioning of Apt-PBMCs to the immobilized CRP on a substrate. It may validate the hypothesis that Apt-PBMCs would be able to detect target molecules and be aggregated to a specific site. In order to confirm the binding specificity potential of Apt-PBMCs toward a target site, we investigated their trafficking and attachment behavior to the CRP-immobilized sites in a microfluidic channel (Supporting Fig. 2). FAM attached aptamer treated PBMCs (Apt-PBMCs/FAM) emitted green fluorescence after mixing with heparinized, diluted blood confirming the successful capture of the target in the channel. The RBC excluded blood sample flowing in a channel showed the attachment of Apt-PBMCs to the CRP-immobilized sites at the flow speed of 0.01 μm/min (0, 4, or 12 Apt-PBMCs at the sites, Supporting Fig. 2C,D). Since the flow speed in a tested channel was faster than the one in a blood vessel, only a few number of cells were attached to the target sites initially but the increased number of Apt-PBMCs were positioned at the target sites afterwards. Further studies employing a wider microchannel and slower flow would help to confirm the binding specificity potential of Apt-PBMCs *in vitro*.

## Conclusion

In this study, we evaluated the properties and functions of Apt-PBMC complexes for use as live cell based sensors to capture CRP. Our strategy is advantageous because PBMCs freely travel within the bloodstream to arrive at specific inflammatory regions with upregulated CRP. Increased attachment of PBMCs by surface engineering with CRP-specific aptamers is expected to promote proper immune system responses to problematic site-specific inflammation. To verify our strategy, we evaluated the successful bioconjugation of aptamers and other crosslinking agents to live PBMCs without denaturing or damaging the inherent nature of the cells. The optimization of the conjugation steps was confirmed by the successful observation of fluorescence from each ligand attached to the cells at each conjugation step. In particular, we focused on minimizing cell damage by calcium ions in the reaction buffer during the conjugation steps. Calcium ions act as cofactors for both CRP detection and ssDNA aptamer formation^23^. Therefore, we directly treated recombinant CRP with CaCl_2_ for binding, and separated the residual calcium ions by centrifugation to minimize cell damage. Filtered/serum-free RPMI1640 was used for washing and as a reaction buffer to reduce the exposure of the cells to residual positive ions. The use of filtered/serum-free RPMI1640 prevented the effects of conjugation steps from the cells resulting in the increase of cell recovery and survival percentages.

We employed dye-conjugated anti-human CRP monoclonal antibodies to detect Apt-PBMC complexes that captured CRP. The limit of detection observed in our assay would be expanded by adapting other detection approaches to our system, such as aptamer beacons that do not require the detection antibody^3^. In addition to the increase in sensitivity, the conjugation steps for the preparation of the live cell based sensing system would also be reduced without detection antibodies, and this would be beneficial with respect to decreasing the total reaction time^3^,^26^. The concentration and affinity of the aptamers determine the sensitivity of live cell based sensors. Future work to advance the design of aptamers and evaluate their affinity would improve the sensitivity of this technique.

Given that there was no significant reduction in the viability of the Apt-PBMC complexes for 3 days, the live cell based sensor system may be used for studies of cell-mediated flexible immune responses. Given that immune cells have flexible responses that are affected by their microenvironments, our live cell based sensing system may serve as an efficient probe to report these flexible responses within a given amount of time (e.g., monitoring crosstalk between immune cells, variation in phenotypes, and the secretion of different cytokines). The simple capturing assay in our study showed that Apt-PBMCs were able to migrate toward regions of higher concentrations of target CRP and were immobilized. This verifies their potential for *in vivo* sensing applications for CRP in the bloodstream of inflammation animal models. Future studies are needed to validate the sensing capability of live cell based sensors in animal models and to examine the application of Apt-PBMCs for analyses of cell-to-cell communication in immunological responses as well as reporter molecules instead of labeled antibody.

## Additional Information

**How to cite this article**: Hwang, J. *et al*. Aptamer-conjugated live human immune cell based biosensors for the accurate detection of C-reactive protein. *Sci. Rep*. **6**, 34778; doi: 10.1038/srep34778 (2016).

## Supplementary Material

Supplementary Information
