# Supplementary material for: Aptamer-conjugated live human immune cell based biosensors for the accurate detection of C-reactive protein
Source: Sci Rep. 2016 Oct 6;6:34778. doi: 10.1038/srep34778 (PMC5052522; doi:10.1038/srep34778)
Supplement: Supplementary Information [file srep34778-s1.doc]

**Supplementary Information**

**Title:** Aptamer-conjugated live human immune cell based biosensors for the accurate detection of C-reactive protein

**Author:** Jangsun Hwang, Youngmin Seo, Yeonho Jo, Jaewoo Son, and Jonghoon Choi^*^


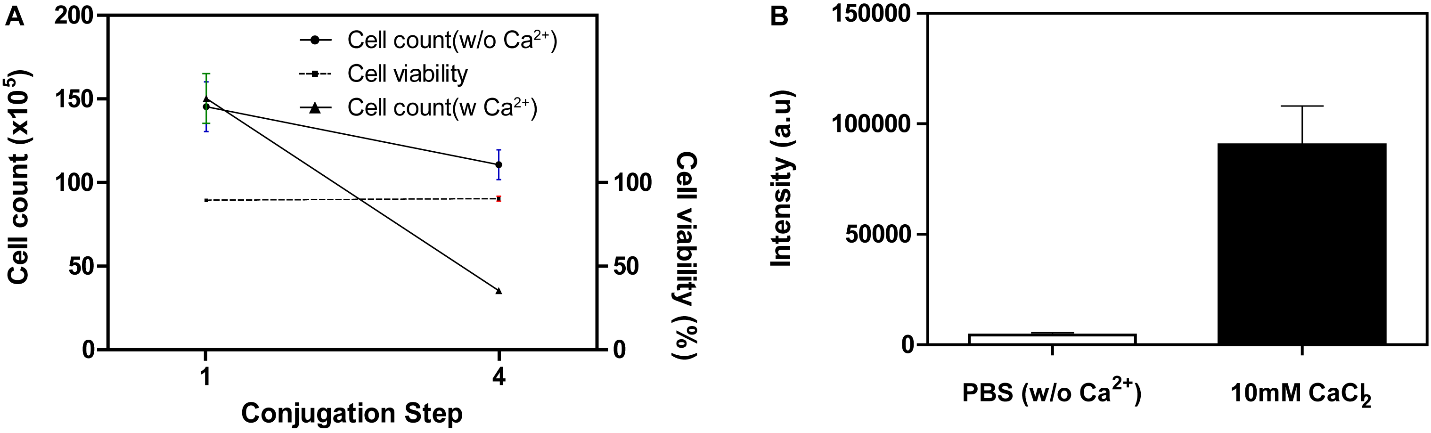


**Supporting Figure 1.** Viability of cells by the conjugation step and the fluorescence intensity with respect to the calcium ions in the solution. **A.** Viability of cells did not change significantly during the steps, but the number of cells counted in the assay changed due to calcium ion in buffer; Only 20% of the initial seeded cells were recovered after conjugation in the buffer solution supplemented with calcium ions, whereas 75% of initial cells were obtained in serum free/filtered-PMI 1640 solution. **B.** Difference in fluorescence intensity toward CRP detection between the samples mixed with PBS containing no calcium ions and the others treated with 10 mM of CaCl_2_ solution (w: with calcium ion, w/o: without calcium ion, Apt-PBMCs: 4 × 10^5^/mL CRP:3 0mg/L).

**
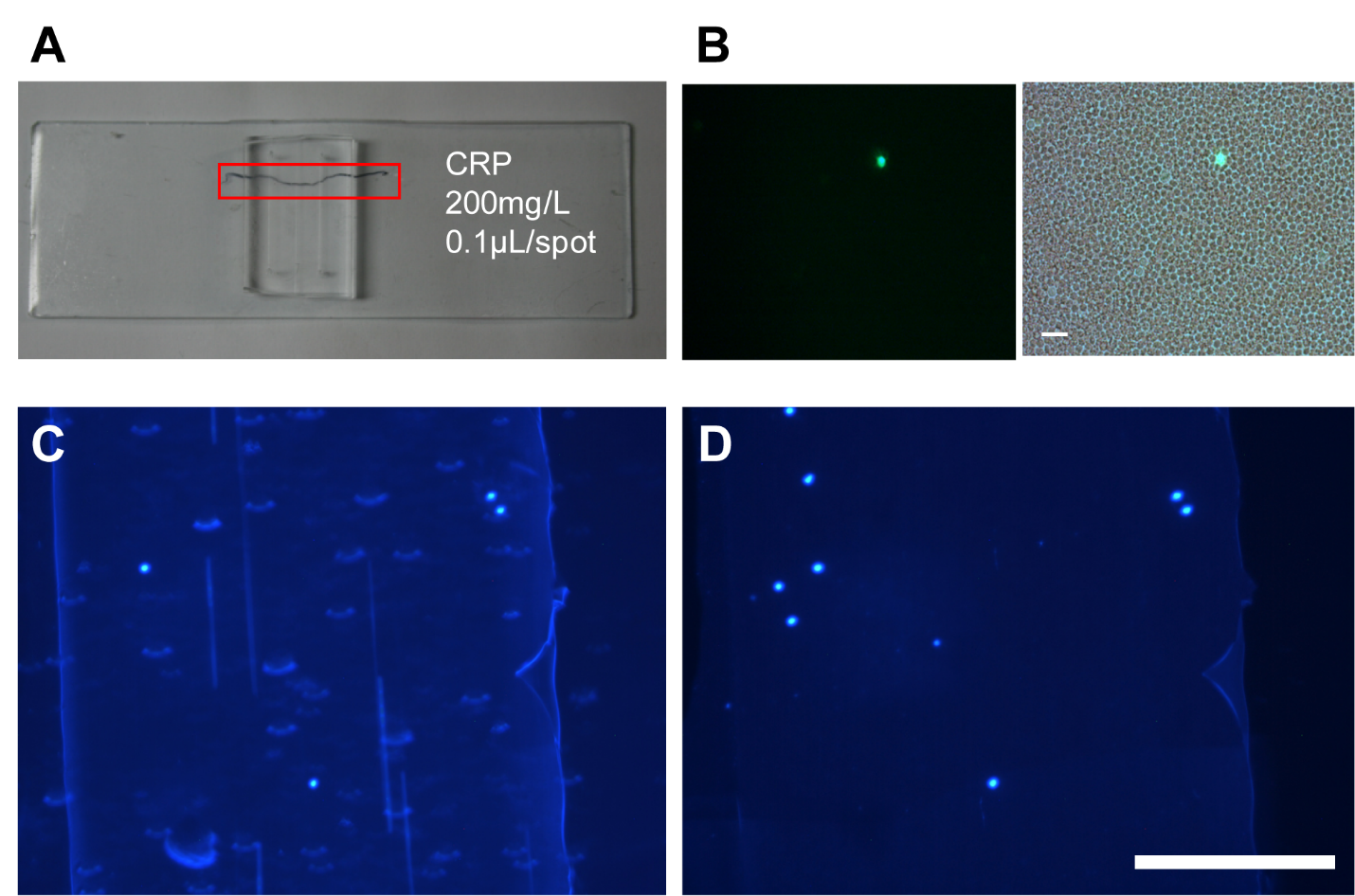
**

**Supporting Figure 2.** Apt-PBMCs attachment assay in microfluidic channels. **A.** CRP spotted PDMS single chip (0.1 µL of CRP was spotted on a plasma treated slide glass by using a microarray spotter, the length of a single channel is 400 µm and the width is 50 µm). **B.** FAM labeled Apt-PBMCs in a sheep whole blood (Apt-PBMC: 5× 10^6^/mL, 1:10000 diluted blood). **C.** Four DAPI stained Apt-PBMCs were observed at the target sites initially **D.** Twelve Apt-PBMCs were observed after 10 hours (flow speed: 0.01 µm/min, Apt-PBMC: 5× 10^3^/mL, Scale bar = 20 µm).


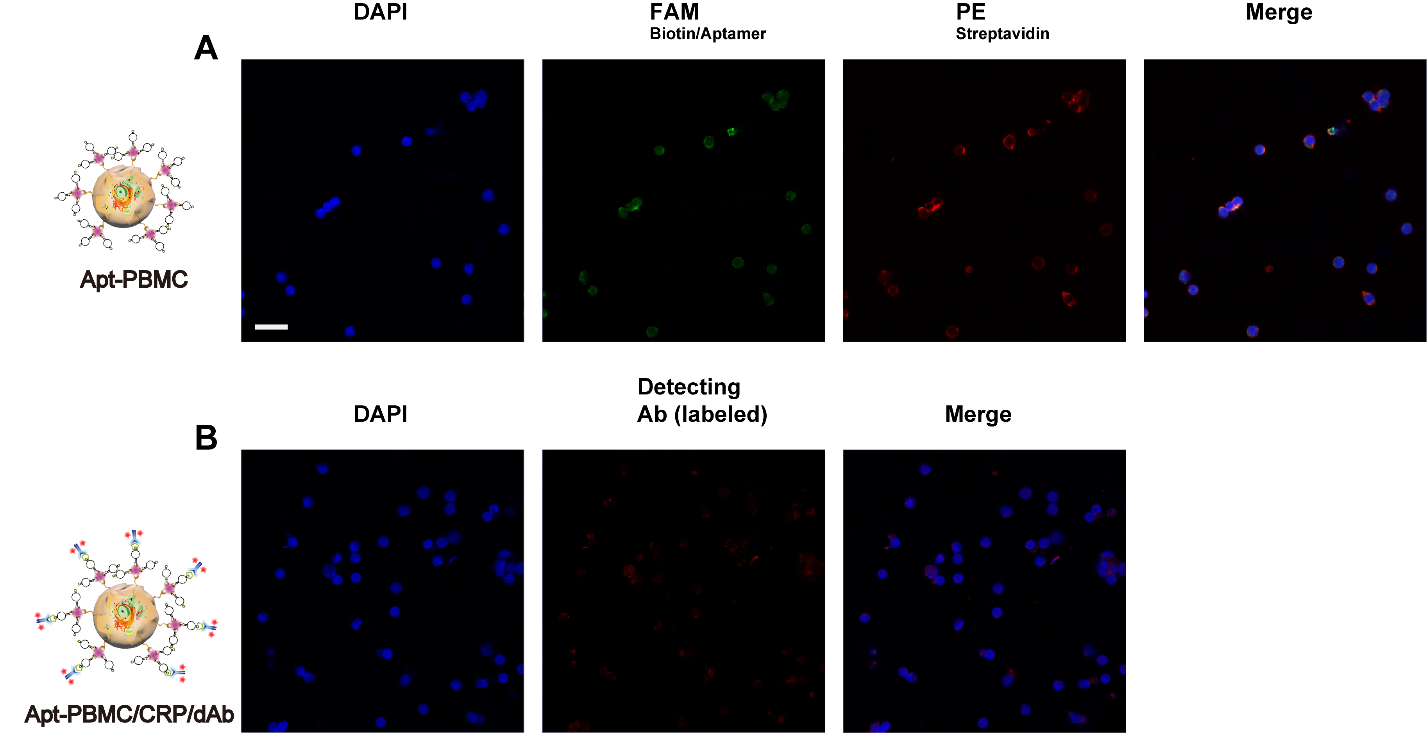


**Supporting Figure 3.** Confocal images of Apt-PBMCs. **A.** Images of each conjugation step (separated and scattered cells were observed). **B.** Fluorescence images of Apt-PBMC complexes capturing CRP (scattered cells were also observed, CRP: 0.1 mg/L, dAb: fluorescence dye labeled antibody, Scale bar = 20 µm).
